# Mental Resilience and Mindfulness in Athletes: A Preliminary Study Across Sports and Experience Levels

**DOI:** 10.3390/sports13100334

**Published:** 2025-10-01

**Authors:** Stanislava Stoyanova, Nikolay Ivantchev, Teodor Gergov, Bilyana Yordanova

**Affiliations:** Department of Psychology, South-West University “Neofit Rilski”, 2700 Blagoevgrad, Bulgaria; nikyart@swu.bg (N.I.); teodor@swu.bg (T.G.); b_iordanova@swu.bg (B.Y.)

**Keywords:** athletes, mental resilience, mindfulness

## Abstract

It is important to study if and how athletes’ mental resilience and mindfulness are connected, because both could promote mental health, as well as facilitate coping with stress and successful athletic performance. It was hypothesized that mental resilience and mindfulness would correlate, and also that mental resilience and mindfulness would differ in both means and correlation strength according to the type of sport. The possible nexus of mental resilience and mindfulness with the longevity of sports practice was also examined. The sample consisted of 177 athletes whose mean age was 21 years old and whose average sports practice lasted 8.7 years. Most of them were professional athletes (*N* = 121, 68.4%). They practiced individual sports (*N* = 85, 48.0%), combat sports (*N* = 47, 26.6%), and team sports (*N* = 45, 25.4%). The Bulgarian adaptations of the Mindful Attention Awareness Scale and the Brief Resilience Scale were used. The athletes’ mental resilience and mindfulness correlated significantly and positively. The athletes’ mental resilience correlated positively with the years of sports practice among the amateur athletes and among the athletes practicing team sports. The athletes’ mindfulness increased with age for the athletes practicing combat sports and team sports. Mindfulness diminished with more years of sports practice for the amateur athletes and for those practicing individual sports. The athletes practicing combat sports had higher mental resilience than those practicing individual sports and those practicing team sports. The male athletes had higher mental resilience than the female athletes. The established positive nexus between the athletes’ mental resilience and mindfulness means that training mindfulness could be a mechanism for strengthening mental resilience, as well as higher mental resilience could facilitate mindful awareness of the present moment and focus attention on what is happening now. The athletes practicing individual sports are those who need more training for enhancing mindfulness and mental resilience. Physical activity and sports participation may contribute to mental health improvement by strengthening mindfulness and mental resilience.

## 1. Introduction

This study aims to examine preliminary the relationship between mental resilience and mindfulness across different sports and levels of experience, with a focus on differences between professional and amateur athletes. It also explores how these traits relate to the duration of sports practice, considering both the benefits of long-term training and the potential effects of age-related cognitive changes. Understanding these links is important, as resilience and mindfulness can support mental health, stress management, and performance.

Physical activity is a well-established factor in health promotion [1] and mental health maintenance [2,3,4], linked to reduced stress, improved mood, higher self-esteem [5,6], and lower risk of depression [7]. Since prevention is easier than treatment, investments in this area have increased [8,9], stimulating interest in sports research.

Physical activity positively affects mental health, improving brain function through increased cerebral blood flow, enhanced neurotransmitter activity (serotonin, dopamine) [10], reduced levels of the stress hormones cortisol and adrenaline, and release of endorphins, which foster well-being, energy, and resilience [9,11]. This contributes to better coping with daily stress and enhances the ability to manage life’s challenges. Taken together, these benefits illustrate the importance of physical activity for mental health that may be enhanced by athletes’ mental resilience and mindfulness.

### 1.1. Resilience and Mindfulness for Improving Mental Health

The World Health Organization defines health as a state of complete physical, mental, and social well-being, not merely the absence of disease or disability [12]. One possible way to prevent health problems is to develop mental resilience and mindfulness, which are often enhanced in physically active individuals such as athletes. Sport serves as a means of increasing mental resilience [13,14]. Athletes tend to be emotionally stable with low levels of neuroticism [15], which predisposes them to mental resilience. Physically active individuals who regularly engage in exercise also demonstrate higher mental resilience [16]. Sport requires physical endurance—for example, mountain running [17]—and mental resilience contributes to this endurance through persistence in pursuing set goals.

According to several studies, athletes generally demonstrated higher levels of mental resilience than individuals working in certain professional sectors—such as economics and information technology [18]—as well as soldiers and students [19], both before and during the coronavirus pandemic. However, during the pandemic, professionals in other fields, including education and law, exhibited greater mental resilience than athletes [20]. This shift is attributed to disruptions in sports training routines and decreased support available to athletes during that period [20]. More resilient athletes employ more adaptive cognitive-emotional coping strategies and perceive fewer barriers to training [21]. Mentally resilient athletes are not easily upset by losses or criticism [22].

Mental resilience—the capacity to adapt positively to adversity—can be conceptualized through the Hardiness Model [23,24] and Cognitive Appraisal Theory [25]. The Hardiness Model emphasizes commitment (active engagement with life), control (belief in one’s capacity to influence events), and challenge (perceiving change or difficulty as an opportunity rather than a threat), which buffer the negative effects of stress and promote adaptive responses [23,24]. Cognitive Appraisal Theory explains resilience as arising from adaptive primary appraisals (interpreting stressors as challenges rather than threats) and secondary appraisals (believing one has sufficient coping resources) [25].

Mental resilience includes perceiving stressful events as challenging and meaningful rather than threatening [23,26,27]; actively transforming stressful situations and adapting to them [28]; being receptive to change [29]; tolerating frustration [30]; having confidence in one’s ability to control the course of one’s life and events; and being engaged in daily activities and committed to one’s pursuits [23,26,29]. In addition to activity, control, and efficacy, other key components of mental resilience include optimism and decisiveness [31].

Mental resilience is a protective resource for maintaining good mental health [32,33], physical health [34], and improving overall health status [35]. Mental resilience is one of the resources enabling athletes to protect their health [36] and serves as an indicator of mental health [33,37]. Mental resilience helps transform the perception of events, making them seem less stressful, while exercise reduces the tension experienced during stressful situations, leading to better health outcomes [24]. Physical exercise combined with hardiness results in greater health benefits than only one of them, though each individually also contributes to health improvement [24].

Another factor related to athletes’ mental health is mindfulness. Mindfulness is a state of wakefulness, alertness, and awareness of the current situation, characterized by a nonjudgmental, self-regulated focus on present experiences and bodily sensations [38,39]. It involves purposefully focusing attention on present sensations, emotional states, needs, and thoughts without judgment of oneself, others, or the situation [40]. Mindfulness means enjoying the present moment rather than dwelling on the past [41] and perceiving unpleasant personal experiences contemplatively instead of reacting emotionally to them [42]. Mindfulness encompasses description, observation, non-evaluative awareness, mindful action, and awareness without reaction [39]. Mindfulness comprises two components: trait mindfulness, which is a stable tendency to maintain nonjudgmental awareness of present experiences, and state mindfulness, a temporary present-moment awareness that fluctuates with context and mood [43].

In stressful situations, trait mindfulness may enhance resilience by fostering constructive appraisals and reinforcing the hardiness dispositions of control and challenge, while state mindfulness may improve attentional clarity and emotional regulation. These processes support effective coping and the maintenance of positive emotions [44]. Resilient individuals use positive emotions to facilitate recovery from stress [45].

Mindfulness is a protective personal resource that reduces stress levels and promotes well-being [39]. Mindfulness training decreases athletes’ depression, perceived stress, and anxiety, while increasing their self-esteem [46] and positive stress-coping behaviors [38]. Attention training can help athletes cope more effectively with stressors, enhance performance, and protect their mental health [36].

### 1.2. Mindfulness Contributing to Better Performance in Sport

Mindfulness contributes to athletic success [38]. Mindfulness training can be used to improve sports performance, particularly in disciplines such as table tennis, archery, and martial arts [47], as well as to enhance the performance of ice hockey teams [48]. Mindfulness practices benefit athletes in both competitive and training contexts by reducing anxiety, stress, and distractions often encountered during competitions, while also fostering greater focus, flow-state experiences, and mind–body connectivity [47]. Mindfulness training enhances athletes’ flow state—characterized by strong concentration during competitions, the elimination of irrelevant thoughts and emotions, the integration of tasks, and the ability to continue competing smoothly even in challenging situations—and this effect is further strengthened by athletes’ resilience [49].

Mindfulness training programs may benefit athletes prone to choking (abnormal performance on the field) by alleviating anxiety, enhancing mental well-being, and optimizing performance outcomes during competitions [50]. Both mindfulness and mental resilience in athletes are negatively correlated with perceived stress and anxiety following choking episodes [50]. Resilience, mindfulness, and consistent sleep routines are also associated with optimizing sports performance and reducing the risk of sport-related injuries [38].

### 1.3. Mental Resilience Contributing to Better Performance in Sport

Mental resilience is important for athletes due to its impact on both health and performance. Enhancing athletes’ mental resilience can improve sports performance, mental health, and coping skills [36], while also promoting overall well-being. Performance and satisfaction are influenced by mental toughness [51]. Individuals with high mental resilience report greater life satisfaction [26], whereas those with lower resilience experience higher levels of burnout [29].

Resilience contributes to athletic success by promoting problem-focused coping with hardship—such as seeking information and support, addressing the source of stress, or removing oneself from the stressful situation—while reducing reliance on emotion-focused coping strategies like venting emotions and behavior disengagement, and by maintaining persistence in sports participation during adversity [38].

### 1.4. Relationships Between Mental Resilience and Mindfulness

Mindfulness correlates positively with mental toughness [38,52]. Athletes with higher levels of mindfulness can detach from negative psychological states more quickly, demonstrate stronger cognitive reappraisal skills, show greater mental toughness in training and competition, and display improved distress tolerance [52]. Both physical exercise and mindfulness contribute to increased mental resilience [53]. Mindfulness fosters overall mental resilience [47].

Mindfulness is related to mental resilience, as it positively influences life satisfaction, positive affect, and emotional regulation [40]. An eight-week mindfulness-based intervention increased mental resilience, self-confidence, and emotional regulation in elite football players aged 16–32, with stronger effects observed in those with an internal locus of control compared to those with an external locus of control [54].

No statistically significant differences in mindfulness have been found between athletes practicing individual and team sports [55]. However, athletes differ in mental resilience depending on the type of sport practiced [22]. Mental resilience is higher in field-striking games—where players score points by hitting an object and running to designated areas on the court or by obstructing opponents—than in target-shooting games, net/wall games, and games involving invasion of opponents’ territory to score points or goals [22].

Physical activities create opportunities for social support, further strengthening resilience [56]. Participation in group sports provides chances to build broad social connections and support networks that serve as sources of mental resilience.

Achievements in various activities—physical, sporting, or work-related—influence resilience [9]. Higher mental toughness is observed in athletes with better sports results across different disciplines, regardless of gender, age, or skill level, except in equestrian and Alpine skiing, which may suggest lower mental resilience among those participating in certain individual sports [57]. Mindfulness training significantly improves resilience in elite athletes [58] and amateur basketball players [59].

The connection between mental resilience and mindfulness has been established in scientific literature [38,47,52,54], but this relationship should be further examined among both amateur and professional athletes, as well as across different sport types and varying lengths of sports experience. Investigating this connection across diverse athletic backgrounds—such as sport type, competitive level, and experience—is important because psychological demands differ by sport, and the competitive level influences stress and coping strategies. Different sports have varying psychological demands on athletes [60,61]. For example, individual sports often require greater self-reliance, relying primarily on internal resources for mental resilience, whereas team sports involve interpersonal dynamics and communication, drawing on external resources. Combat sports combine both self-reliance and interactions, potentially requiring higher levels of mental resilience and mindfulness to manage anxiety and maintain focus.

Athletes at different competitive levels—amateur and professional—face distinct stressors [62]. Professional athletes may encounter intense media scrutiny and heightened performance pressure, whereas amateur athletes must balance sport with other responsibilities, such as academics or work. Both contexts require mental resilience and mindfulness to cope effectively. Amateur athletes, who may still be developing coping strategies, could benefit from mindfulness to strengthen resilience, while professional athletes might use mindfulness to fine-tune performance or prevent burnout. Examining how the length of sport experience shapes the relationship between mindfulness and mental resilience can help determine whether mindfulness should be introduced early in an athlete’s career or adapted to specific career stages. Understanding the interactions between mental resilience and mindfulness across athlete profiles could enhance the effectiveness of psychological support services.

In sport-specific settings, mental resilience and mindfulness are expected to interact in a dynamic and mutually reinforcing way. Mindfulness—the practice of maintaining present-moment awareness with acceptance [44]—can enhance an athlete’s ability to observe stressful or challenging situations without immediate judgment or emotional reactivity. This mindful awareness may support the development of mental resilience by enabling athletes to better regulate emotions, maintain focus under pressure, and recover more effectively from setbacks.

Conversely, higher levels of mental resilience may facilitate deeper engagement with mindfulness practices, as resilient athletes tend to demonstrate greater persistence [63], adaptive coping strategies such as problem-focused coping, optimism, and seeking social support [64], and openness to learning from experiences [65]. Together, these capacities help athletes navigate the unique psychological demands of their specific sports, whether it involves sustained concentration in individual sports, quick adaptability in team sports, or anticipating opponents’ movements in combat sports.

Overall, the interaction between mental resilience and mindfulness may contribute to enhanced performance, well-being, and long-term athletic development within the specific demands of each sport. Studying the relationship between mindfulness and mental resilience across diverse athlete backgrounds ensures that psychological interventions are strategically designed to address the nuanced needs of athletes based on their sport type, competitive level, and experience.

### 1.5. Scientific Gap

While mental resilience and mindfulness have each been studied in relation to athletic performance (for example, [38]) and well-being (for example, [36]), existing research tends to focus on single constructs in isolation in specific sports (for example, {22]) or within specific populations like elite athletes (for example, [54,58] and amateur basketball athletes [59], rather than exploring their interrelationship across different types of sports and among both amateur and professional athletes. While mindfulness training is shown to enhance mental resilience in both elite [58] and amateur athletes [59], there is a lack of research directly comparing these relationships across different sport types. There is limited knowledge about how mental resilience and mindfulness are linked in athletes across diverse sporting contexts or whether these relationships differ between professionals and amateurs, given variations in training demands, performance pressure, and resources for psychological support. There is limited cross-sport analysis of the relationship between mindfulness and mental resilience. Comparative studies directly contrasting the mindfulness–resilience relationship between professionals and amateurs across various sports are lacking.

Little is known about (a) whether the relationship between mindfulness and resilience differs across sport types and competitive levels (professional vs. amateur), and (b) how the duration of sports practice and age-related cognitive changes are connected with mindfulness and mental resilience in athletes. There is limited knowledge of what the role of duration of practice is—whether long-term participation enhances mindfulness and resilience, or whether age-related declines in attention and cognitive flexibility alter these associations. It remains unclear how the duration of sports participation and age-related cognitive changes relate to mindfulness and resilience.

This scientific gap is important because understanding the nuanced interaction between resilience and mindfulness across sport type, competitive level, and athletic lifespan could inform more targeted mental skills training programs, supporting both performance and well-being for athletes at all stages. Addressing this gap will clarify how psychological skills evolve across the athletic career span, informing tailored interventions for athletes at different levels.

### 1.6. Hypotheses

The literature review provides some reasons to hypothesize that mental resilience and mindfulness are correlated across different types of sports, with a stronger correlation expected among professional athletes compared to amateur athletes. We also expect that mental resilience will vary depending on the type of sport practiced.

Additionally, we intend to examine the nexus of mental resilience and mindfulness with the longevity of sports practice. Higher mental resilience is expected among athletes with longer sports experience, practice, and training, as prolonged engagement in sport requires endurance, persistence, and self-control. Mindfulness is also expected to be related to the duration of sports practice, since more experienced athletes may have developed greater focused attention to present stimuli. However, it should be noted that athletes with more years of sports practice may also be older and could experience some decline in cognitive functioning.

## 2. Materials and Methods

The study was conducted in 2023–2024 using both face-to-face and online methods. Questionnaires were distributed in person to some participating athletes, who completed and returned them directly to the researcher, while others received a link to complete the questionnaires online. Inclusion criteria required participants to be adult athletes (18 years or older) with at least one year of sports practice who voluntarily agreed to participate. The study was conducted in accordance with the Declaration of Helsinki [66].

### 2.1. Sample

Assuming a significance level of alpha = 0.05 and anticipating a moderate correlation between mental resilience and mindfulness (*r* = 0.30), a sample size of 139 athletes is required to achieve a statistical power of 0.95 [67]. Only data from participants with complete responses (no missing data) were included in the statistical analysis.

The sample consisted of 177 athletes, the majority of whom were male (*N* = 95, 53.7%), with fewer female athletes (*N* = 82, 46.3%). Their ages ranged from 18 to 28 years (*M* = 21; *SD* = 3). They practiced sports for between 1 and 20 years (*M* = 8.7; *SD* = 5.3). Nearly half of the participants (*N* = 87, 49.2%) had ten or more years of sports experience. Most were professional athletes (*N* = 121, 68.4%), while approximately one-third were amateur athletes (*N* = 56, 31.6%). They participated in various sports, including individual sports such as track and field athletics, swimming, gymnastics, winter sports, triathlon, modern pentathlon, wind sports, etc. (*N* = 85, 48.0%); combat sports such as boxing, sports wrestling, judo, taekwondo, karate, etc. (*N* = 47, 26.6%); and team sports such as volleyball, soccer, basketball, field hockey, baseball, etc. (*N* = 45, 25.4%). One variable in the dataset coded each specific sport separately, while another variable categorized the sport type according to the classification by [68] and partly based on [69].

### 2.2. Instruments

The Bulgarian adaptation [70] of the Mindful Attention Awareness Scale (MAAS [71]) for athletes was used. The scale consists of 15 items rated on a 6-point scale [70,71]. Among Bulgarian athletes, MAAS has a Cronbach’s alpha of 0.80 [70]. Higher scores indicate greater present-moment attention and awareness—that is, higher mindfulness [70,71].

The Bulgarian adaptation of the Brief Resilience Scale (BRS) [19], including data from athletes, was used. Originally developed by [35], the BRS measures mental resilience as the ability to recover quickly from stress. It consists of 6 items rated on a 5-point Likert scale, with items 2, 4, and 6 reverse-coded [16,19,35,72,73]. The mix of positively and negatively worded items aims to reduce social desirability [35]. In several Bulgarian studies, Cronbach’s alpha ranges from 0.82 to 0.865 [19,72,74], supporting its reliability as a unidimensional scale [19]. Higher scores indicate greater resilience [73].

Levels of mindfulness and resilience were classified using mean and standard deviation values from the Bulgarian adaptations of the questionnaires [19,71], with low scores defined as below M − SD and high scores as above M + SD.

Some social and demographic data were collected to enable group comparisons of the athletes’ responses.

### 2.3. Data Analysis

Data were analyzed using SPSS 23 and JASP 0.19.3.0. Descriptive statistics were calculated. The Shapiro–Wilk test assessed normality of score distributions. For variables that did not meet the normality assumption, Spearman’s rho was used to examine correlations. Group comparisons were conducted using the non-parametric Mann–Whitney U test and chi-square analysis.

Correlation coefficients, adjusted for sample size, were compared with Soper’s calculator [75] to evaluate relationship strength across subgroups of unequal size.

Following Johanson and Brooks [76], only groups with ≥12 participants were compared, as this size is sufficient to detect strong effects (*r* = 0.70) with 80% power at α = 0.05 [67] (p. 102). Sensitivity analysis was conducted by re-running the main analyses excluding each of the smaller sport groups (soccer, *N* = 13; volleyball, *N* = 18; and swimming, *N* = 19). A minimum of 28 participants was required to detect moderate effects (*r* = 0.50) with 80% statistical power at α = 0.05 [67], a threshold exceeded by the next group (track and field, *N* = 31; see Table 1). Findings from smaller subgroups should be considered preliminary.

In multiple correlation analyses between mindfulness and resilience across athlete subgroups (all > 12 participants; *N* = 177), we controlled for inflated Type I error using the Holm–Bonferroni method [77] and for false discovery rate using the Benjamini–Hochberg procedure [78,79].

Statistical power was assessed to ensure adequate subgroup sizes, with effect sizes and confidence intervals reported for precision and interpreted following [80].

Bayesian statistics were also used to quantify evidence for or against null and alternative hypotheses. A Bayes Factor (*BF*_10_) > 3 indicates some or moderate evidence for the alternative hypothesis, >10 indicates strong evidence, and >30 very strong evidence [81,82]. Conversely, *BF*_01_ > 3, >10, and >30 indicate increasing levels of evidence in favor of the null hypothesis [81,82].

## 3. Results

### 3.1. Descriptive Statistics

Athletes showed moderate levels of both resilience (76.8%) and mindfulness (49.7%). High and low resilience were similarly distributed (12.4% vs. 10.7%), whereas low mindfulness was more frequent than high (31.1% vs. 19.2%). Compared with Bulgarian norms [19,71], mindfulness scores (3.765 ± 0.846) and resilience scores (3.352 ± 0.753) among participating athletes were slightly lower. Shapiro–Wilk tests showed non-normal distributions for MAAS (Shapiro–Wilk = 0.976, *df* = 177, *p* = 0.004), BRS (Shapiro–Wilk = 0.980, *df* = 177, *p* = 0.012), age (Shapiro–Wilk = 0.474, *df* = 177, *p* < 0.001), and years of sports experience (Shapiro–Wilk = 0.891, *df* = 177, *p* < 0.001).

### 3.2. Correlations

The athletes’ mental resilience and mindfulness were significantly and positively correlated in the overall sample, as well as among professional athletes and those practicing individual sports, swimming, volleyball, and combat sports (see Table 1). The results from small subgroups should be regarded as preliminary. Differences between these correlational coefficients, given their associated sample sizes, were not statistically significant (*p* > 0.05) when compared using a statistical calculator [75], indicating weak to moderate associations between athletes’ mental resilience and mindfulness.

Because some correlation coefficients had large confidence intervals (see Table 1) and to reduce the probability of Type I error due to many correlational tests within one sample, we applied the Holm–Bonferroni method to adjust *p*-values (see Table A5 in Appendix B) and the Benjamini–Hochberg method to control the expected proportion of false positives among rejected hypotheses (see Table A1 in Appendix A). The results indicated that mental resilience and mindfulness were significantly correlated in the overall sample of the studied athletes and among professional athletes (see Table A1 in Appendix A and Table A5 in Appendix B). These findings suggest cautious interpretation of the correlations between mental resilience and mindfulness among athletes practicing individual sports, swimming, volleyball, and combat sports.

Bayesian statistics provided strong evidence for a correlation between mental resilience and mindfulness in the overall sample of athletes (*BF*_10_ = 16.932, *BF*_01_ = 0.059). Moderate evidence was found for professional athletes (*BF*_10_ = 9.261, *BF*_01_ = 0.108) and for those practicing swimming (*BF*_10_ = 3.968, *BF*_01_ = 0.252). Weak evidence was observed for athletes practicing individual sports (*BF*_10_ = 2.994, *BF*_01_ = 0.334), combat sports (*BF*_10_ = 1.710, *BF*_01_ = 0.585), and volleyball (*BF*_10_ = 2.881, *BF*_01_ = 0.347). These findings suggest that correlations between mental resilience and mindfulness among the athletes practicing individual sports, volleyball, and combat sports should be interpreted with caution.

The studied professional athletes and amateur athletes did not differ statistically significantly in age (Mann-Whitny *U* = 3272, *p* = 0.704) or years of sports training (Mann-Whitny *U* = 3359, *p* = 0.927). However, amateur athletes were significantly more frequently female than expected, whereas professional athletes were significantly more frequently male than expected (χ^2^ _(*df* = 1,_ *_N_*_= 177)_ = 12.842, *p* < 0.001, Phi = 0.269). Therefore, partial correlations between mental resilience and mindfulness controlling for gender were computed. Results showed significant positive correlations between mental resilience and mindfulness for both amateur athletes (*r*_(53)_ = 0.269, *p* = 0.047) and professional athletes (*r*_(118)_ = 0.257, *p* = 0.005).

Athletes’ mental resilience did not correlate significantly with age, either in the overall sample or within specific subgroups of athletes (see Table 2). This finding suggests that mental resilience remains stable between the ages of 18 and 28, the age range of the athletes studied.

Mindfulness tended to increase with age among the athletes in combat sports, team sports, and especially volleyball (see Table 3). However, the narrow age range (18–28 years) warrants cautious interpretation of these findings. Differences between these correlational coefficients, given their associated sample sizes, were not statistically significant (*p* > 0.05) when compared using a statistical calculator [75], indicating moderate correlations between mindfulness and age in these groups of athletes.

Because some correlation coefficients had large confidence intervals (see Table 3) and to reduce the probability of Type I error from multiple correlational tests within a single sample, we applied the Holm–Bonferroni method to adjust *p*-values (see Table A6 in Appendix B) and the Benjamini–Hochberg method to control the expected proportion of false positives among rejected hypotheses (see Table A2 in Appendix A). After adjusting significance levels, the results indicated that mindfulness did not correlate significantly with age in the overall sample or within any athlete subgroups (see Table A2 in Appendix A and Table A6 in Appendix B). These findings warrant cautious interpretation of correlations between mindfulness and age in athlete subgroups.

Bayesian analysis provided strong evidence for a correlation between mindfulness and age among the athletes practicing combat sports (*BF*_10_ = 10.850, *BF*_01_ = 0.092), moderate evidence for athletes practicing volleyball (*BF*_10_ = 5.789, *BF*_01_ = 0.173), and weak evidence for athletes practicing team sports (*BF*_10_ = 2.365, *BF*_01_ = 0.423). These findings warrant cautious interpretation of correlations between mindfulness and age in some athlete subgroups.

Mental resilience correlated positively with years of sports practice among the soccer players, amateur athletes, and athletes practicing team sports (see Table 4). Bayesian analysis provided strong evidence for these associations between mental resilience and years of sports practice in team sports (*BF*_10_ = 10.925, *BF*_01_ = 0.092), especially in soccer (*BF*_10_ = 44.720, *BF*_01_ = 0.022), and among amateur athletes (*BF*_10_ = 11.433, *BF*_01_ = 0.087).

All soccer players in the study were male, with nearly equal numbers of amateurs (*N* = 6) and professionals (*N* = 7). The team sports group included both amateurs (*N* = 19) and professionals (*N* = 26).

The difference between the correlational coefficients for athletes practicing team sports and those practicing soccer, compared using a statistical calculator that accounts for their sample sizes [75], was statistically significant (*z* = −2.025, *p* = 0.027). This indicates a moderate correlation between mental resilience and years of sports practice in team sports and a strong correlation in the soccer subgroup. Similarly, the difference between the correlational coefficients for amateur athletes and athletes practicing soccer, compared using a statistical calculator that accounts for their sample sizes [75], was statistically significant (*z* = −2.340, *p* = 0.019). This indicates a moderate correlation between mental resilience and years of sports practice in amateurs and a strong correlation in soccer players. However, the difference between the correlational coefficients of team sports athletes and amateur athletes, compared using a statistical calculator that accounts for their sample sizes [75], was not statistically significant (*p* > 0.05). This indicates moderate correlations between mental resilience and years of sports practice in both groups.

Due to large confidence intervals of some correlation coefficients (see Table 4) and to reduce the risk of Type I error from multiple correlational tests within the same sample, we applied the Holm–Bonferroni method to adjust *p*-values (see Table A7 in Appendix B) and the Benjamini–Hochberg method to control the expected proportion of false positives among rejected hypotheses (see Table A3 in Appendix A). After adjustment of significance levels, mental resilience correlated statistically significantly with years of sports practice among soccer players, amateur athletes, and team sports athletes according to the Benjamini–Hochberg method (see Table A3 in Appendix A), but only among soccer players according to the Holm–Bonferroni method (see Table A7 in Appendix B). These findings suggest cautious interpretation of the correlations between mental resilience and years of sports practice in amateur athletes and team sports participants.

Mindfulness decreased with more years of sports practice in the entire sample, as well as among track and field athletes, amateur athletes, and those practicing individual sports (see Table 5). However, the differences between these correlational coefficients, accounting for sample sizes, were not statistically significant (*p* > 0.05) based on comparisons using a statistical calculator [75], indicating weak to moderate correlations between mindfulness and years of sports practice in these groups.

Due to large confidence intervals of some correlation coefficients (see Table 5) and to reduce the risk of Type I error from multiple correlational tests within the same sample, we applied the Holm–Bonferroni method to adjust *p*-values (see Table A8 in Appendix B) and the Benjamini–Hochberg method to control the expected proportion of false positives among rejected hypotheses (see Table A4 in Appendix A). After adjustment of significance levels, mindfulness did not correlate statistically significantly with years of sports practice in the whole sample of athletes or any subgroups (see Table A3 in Appendix A and see Table A7 in Appendix B). These findings suggest cautious interpretation of the correlations between mindfulness and years of sports practice in the total sample, track and field athletes, amateur athletes, and those practicing individual sports.

Bayesian statistics indicated moderate evidence supporting the alternative hypothesis of a statistically significant correlation between mindfulness and years of sports practice among athletes practicing individual sports (*BF*_10_ = 3.051, *BF*_01_ = 0.328) and track-and-field athletes (*BF*_10_ = 4.788, *BF*_01_ = 0.209). Weak evidence was also found for a statistically significant correlation between mindfulness and years of sports practice among the amateur athletes (*BF*_10_ = 1.430, *BF*_01_ = 0.699). Bayesian statistics provided weak evidence supporting the null hypothesis about the lack of significant correlation between mindfulness and years of sports practice in the whole sample of athletes (*BF*_10_ = 0.801, *BF*_01_ = 1.249). These findings suggest cautious interpretation of the correlations between mindfulness and years of sports practice, particularly among the amateur athletes and the overall sample of athletes without differentiation by the type of sport.

Sensitivity analysis was also conducted by re-running the main analyses excluding each of smaller sport categories (soccer, volleyball, and swimming), and the results remained consistent with the original findings reported here.

### 3.3. Group Differences

There were not any statistically significant differences in mindfulness between the athletes practicing individual sports and combat sports (Mann–Whitney *U* = 1821.500, *p* = 0.403), between team sports and individual sports (Mann–Whitney *U* = 1861.500, *p* = 0.803), or between team sports and combat sports (Mann–Whitney *U* = 973.000, *p* = 0.509).

The athletes practicing combat sports (mean rank = 82.09, *M* = 3.68, *SD* = 0.74) had higher mental resilience (Mann–Whitney *U* = 1265.000, *p* < 0.001, η^2^ = 0.092, i.e., intermediate effect size; very strong evidence in support of the alternative hypothesis as *BF*_10_ = 151.718 and *BF*_01_ = 0.007) than those practicing individual sports (mean rank = 57.88, *M* = 3.19, *SD* = 0.68). Similarly, the athletes practicing combat sports (mean rank = 52.46, *M* = 3.68, *SD* = 0.74) had higher mental resilience (Mann–Whitney *U* = 777.500, *p* = 0.028, η^2^ = 0.052, i.e., small to intermediate effect size; weak evidence in support of the alternative hypothesis as *BF*_10_ = 2.073 and *BF*_01_ = 0.483) than those practicing team sports (mean rank = 40.28, *M* = 3.32, *SD* = 0.80). There were not any statistically significant differences in mental resilience between the athletes practicing team sports and those practicing individual sports (Mann–Whitney *U* = 1739.500, *p* = 0.395).

There were not any statistically significant gender differences in mindfulness (Mann–Whitney *U* = 3573.000, *p* = 0.343). The male athletes (mean rank = 100.03, *M* = 3.49, *SD* = 0.76) had higher mental resilience (Mann–Whitney *U* = 2837.500, *p* = 0.002, η^2^ = 0.055, i.e., small to intermediate effect size; moderate evidence in support of the alternative hypothesis as *BF*_10_ = 3.507 and *BF*_01_ = 0.285) than the female athletes (mean rank = 76.10, *M* = 3.20, *SD* = 0.72) participating in the study.

There were not any statistically significant differences in mindfulness (Mann–Whitney *U* = 3060.000, *p* = 0.301) or mental resilience (Mann–Whitney *U* = 3054.500, *p* = 0.291) between professional and amateur athletes.

## 4. Discussion

The positive link between athletes’ mental resilience and mindfulness suggests that mindfulness training may strengthen resilience, while greater resilience may enhance present-moment awareness.

In this sample, mental resilience positively correlated with years of sports practice among soccer players, amateurs, and team sport athletes, with Bayesian analyses supporting these associations. Correlations were strongest in soccer, but small subgroup sizes and wide confidence intervals warrant caution. After multiple-test corrections, significant correlations remained for soccer under both Holm–Bonferroni and Benjamini–Hochberg methods, and for amateurs and team sports only under the less conservative Benjamini–Hochberg method. These findings point to a potential association between mental resilience and sports experience, possibly reflecting accumulated resources such as social support in team sports, recognition in soccer, or healthy lifestyle among amateur athletes, though causal inferences cannot be drawn from this cross-sectional study.

Male athletes showed higher resilience than females, consistent with evidence that women experienced stronger emotions [40] and higher rates of depression and anxiety [38], while men received greater social freedom during socialization [16]. Similar gender differences in resilience have been observed in both general [16,83] and athletic samples [68].

No significant correlation emerged between athletes’ mental resilience and age, consistent with some previous scientific findings [57] supporting the view that mental resilience is a relatively stable construct [36]. However, in team sports—particularly soccer—longer sports practice was linked to higher resilience, likely due to greater social support, which protects against anxiety and depression [38]. Additionally, practicing combat sports appears beneficial for maintaining high mental resilience, likely due to good physical fitness, self-confidence in one’s ability to defend oneself, and positive appraisal of success achieved in sport. Overall, sports participation enhances mental resilience [13,53,73] and reduces stress [24], with the effect most evident among team sport athletes, soccer players, and amateurs.

In this cross-sectional study, mindfulness correlated positively with age in combat sports, team sports, and especially volleyball, but these associations lost significance after correction for multiple comparisons, suggesting sampling variability. Bayesian analyses indicated strong evidence in combat sports, moderate in volleyball, and weak in team sports, though wide confidence intervals, inconsistent evidence across methods, and the exploratory nature of subgroup analyses call for caution. These findings tentatively suggest that maturation and the demands of certain sports may foster greater present-moment awareness for the athletes practicing combat sports and some team sports, consistent with evidence that aging promotes mindful qualities such as observing (especially women), describing, acting with awareness and paying attention to one’s actions (especially men), accepting one’s inner experiences without judging emotions as bad or inappropriate, and contemplating without reacting to internal experiences [40].

Mindfulness tended to decline with more years of practice in the overall athlete sample, as well as in individual sports, among track and field athletes, and amateur athletes, though evidence weakened after correction for multiple comparisons and Bayesian analysis, so these results require cautious interpretation. Due to the study’s cross-sectional design, no causal inferences can be drawn regarding the relationship between mindfulness and years of sports practice.

The negative association between athletes’ mindfulness and years of sports practice cannot be explained by age, since older athletes in combat and team sports showed higher mindfulness, aligning with broader population trends [84]. The observed decrease in mindfulness with increased years of sports practice may reflect both the effects of age and experience. Future studies with broader age ranges or longitudinal designs would be better suited to isolate these influences. This pattern suggests that extended sports engagement does not necessarily maintain or enhance mindfulness and could be influenced by factors such as routine-driven training, performance pressure, or cognitive fatigue accumulated over years of practice. Athletes in individual sports such as track and field athletics and amateurs appeared especially prone to reduced present moment focus with more years of sports practice, possibly due to distraction by past experiences, future expectations, or strong emotions.

Athletes in individual sports showed lower resilience than those in team and combat sports, possibly due to differences in self-regulation and attentional focus or disregarding information they consider irrelevant to successful performance in the moment Further research on athletes’ self-regulation, coping strategies, and decision-making is needed to clarify their links to mindfulness and resilience.

Athletes practicing individual sports are those who may benefit most from additional training in mindfulness and mental resilience. Future directions for enhancing mental resilience are setting clear goals and committing to them; viewing challenges as opportunities for growth; being flexible and open to change; cultivating a positive mindset; using positive self-talk through positive affirmations that remind one of strengths and success; practicing some stress management techniques such as mindfulness, meditation, deep breathing exercises, and gratitude to focus on positivity; seeking support; maintaining a healthy lifestyle, etc. [18]. Mindfulness is typically developed through meditation and exercises designed to improve focused attention and promote relaxation [54,85].

Physical activity supports a healthy lifestyle and improves health [24,86], though risks exist with overtraining, injuries, misuse of pharmaceuticals, eating disorders, and other factors [87]. The negative link between mindfulness and years of practice appeared only in amateurs, not professionals, suggesting it is not due to excessive training. In amateurs, more years of practice were associated with greater resilience, which may protect mental health. Overall, regular but balanced training promotes physical and mental well-being, fostering resilience and mindfulness as key protective resources.

The limitations of this study include the small sample sizes of some athlete subgroups, unequal group sizes in comparisons, and its cross-sectional design rather than a longitudinal or intervention-based approach, which would better establish the dynamics of the studied phenomena. However, this study’s goal was not to promote the development of mental resilience and mindfulness in athletes, but rather to establish their connection in usual sports practice.

The groups of athletes compared in this study were selected based on recommendations for minimum required sample sizes [76] and power analysis [67]. While many of the observed correlations were small to medium in magnitude, subgroup comparisons included a minimum of 12 participants, consistent with guidelines for preliminary or exploratory research [76]. This sample size was considered sufficient to detect strong effects (*r* = 0.70) with 80% statistical power at α = 0.05 [67]. Although smaller subgroup sizes reduce power to detect weaker associations, the goal was to ensure adequate representation across multiple categories while balancing practical constraints such as participant availability and subgroup diversity. Therefore, results from smaller subgroups should be viewed as preliminary. Additionally, a sensitivity analysis was performed by re-running the main analyses after excluding each of smaller sport categories (soccer *N* = 13, volleyball *N* = 18, and swimming *N* = 19). Since a group size of 28 was sufficient to detect a moderate effect (*r* = 0.50) with 80% statistical power at α = 0.05 [67], the results remained consistent with those from the whole sample.

Several measures were taken to assess the extent to which we could rely on the results of the study and provide nuanced interpretation of the findings. These included reporting confidence intervals, effect sizes, and Bayesian statistics indicating the probability of support for the alternative hypotheses. Additionally, significance levels were adjusted in the correlational analyses to account for multiple hypothesis testing, thereby reducing the risk of Type I error through the Holm–Bonferroni and Benjamini–Hochberg methods. This approach enables cautious inference even when statistical power may be limited and promotes transparency regarding the strength and reliability of the observed effects. Furthermore, comparing differences between two correlation coefficients—adjusted for their respective sample sizes [75]—allows assessment of the relative strength of relationships between variables across participant subgroups, even when subgroup sizes are unequal.

Another limitation of the study may be related to the exclusive use of self-report questionnaires, as previous research has shown that people tend to overestimate their own traits when several traits were measured with self-report questionnaires rather than objective measures [88]. The present study measured only two personality attributes, and some previous findings were replicated about the positive correlation between athletes’ mindfulness and mental resilience [38,52], higher mental resilience in male athletes consistent with general population data [16,83], and the absence of differences in mindfulness according to the types of sport [55], which may indicate honest responding.

Both the Brief Resilience Scale (BRS) and the Mindful Attention Awareness Scale (MAAS) rely on self-report, making them susceptible to social desirability bias, particularly in athletic populations where mental toughness, composure, and attentional focus are culturally valued traits. Athletes may consciously or unconsciously overreport mindfulness or resilience to align with perceived performance ideals or coaching expectations. This bias could artificially inflate the observed correlations between mindfulness and resilience by introducing common method variance—the tendency for relationships between constructs to appear stronger or weaker when both are measured using the same assessment format [89]. Consequently, the observed association may partially reflect response style rather than the true underlying relationship between these constructs. To reduce common method variance, this study employed several procedures, including ensuring anonymity [90,91], using reverse-coded items [92], and varying the number of possible answers per scale [91,92]. Additionally, the mix of positively and negatively worded items of Brief Resilience Scale aims to reduce social desirability [35].

To strengthen construct validity and reduce common-method bias, future research may incorporate multi-method triangulation by collecting data from multiple sources [90]. This could include observer ratings—such as coach or teammate assessments of composure, adaptability, and attention focus—along with behavioral measures (for example, change in performance following positive or negative audience reactions during a sports match, or after feedback from a coach or teammates, as indicators of mental resilience; fast and accurate detection of subtle changes in visual scenes, or precise description of the current situation, as indicators of mindful attention). Physiological indices—such as skin conductance response, heart rate variability, or cortisol levels following stress—could be used to capture non-self-reported indicators of mindfulness and mental resilience.

By integrating self-report data with behavioral, observational, and physiological measures, future studies could better distinguish genuine relationships between mindfulness and resilience from artifacts of measurement format or social desirability pressures. Using multiple methods and data sources may improve both the accuracy and validity of findings. Additionally, studying diverse samples of athletes from different cultures could help establish more precisely the relationship between mental resilience and mindfulness across various sports and training durations. In this regard, our findings replicate those of [38,47,52,53], providing evidence of honest responding and minimal common-method bias.

## 5. Conclusions

This study found a positive relationship between mental resilience and mindfulness differentiated by sport type, gender, age, and duration of sport practice. In this cross-sectional study of athletes, mental resilience and mindfulness were positively correlated in the overall sample and among professional athletes, with Bayesian analyses providing strongest support for these groups. Similar associations observed in some sport-specific subgroups were weaker and exploratory. Professional and amateur athletes did not differ significantly in age or years of training, but they differed in gender distribution; after controlling for gender, the positive association persisted in both groups. These findings indicate a potential relationship between mental resilience and mindfulness, but the results—particularly for subgroups—should be interpreted with caution, and no causal inferences can be made.

Mental resilience showed no correlation with age within 18–28 years, suggesting stability. However, longitudinal studies are needed to clarify the developmental dynamics of mental resilience. Moreover, mindfulness tended to increase with age in combat and some team sports demanding short bursts of maximal effort, quick reaction times, concentration and emotional control under pressure, as well as agility, coordination, and precise body positioning and timing. Among amateurs, resilience rose with years of sports practice, while mindfulness declined, highlighting a need for mindfulness training.

Establishing connection between mental resilience and mindfulness across sports and experience levels may highlight those who need to strengthen them, that is crucial for both performance and mental health. Mental resilience allows athletes to cope with adversity, recover from setbacks, regulate emotions, and sustain performance under pressure, while mindfulness supports present-moment focus and concentration. Physical activity and sports participation may improve mental health by strengthening both mindfulness and mental resilience. Integrating mindfulness training with programs aimed at expanding coping strategies may further reinforce athletes’ mental resilience, leading to improved performance, health, and overall well-being.

## Figures and Tables

**Table 1:** Correlations between mental resilience and mindfulness.

Groups of Participants	Spearman’s Rho (with Confidence Intervals)	*p*	*N*
overall sample of athletes	0.235 (0.090 ÷ 0.369)	0.002 *	177
athletes in individual sports	0.255 (0.044 ÷ 0.444)	0.019 *	85
athletes in combat sports	0.312 (0.017 ÷ 0.543)	0.033 *	47
athletes in team sports	0.282 (−0.013 ÷ 0.531)	0.061	45
athletes in swimming	0.517 (0.082 ÷ 0.786)	0.023 *	19
athletes in volleyball	0.524 (0.076 ÷ 0.796)	0.026 *	18
track and field athletes	0.028 (−0.330 ÷ 0.378)	0.882	31
athletes in soccer	−0.253 (−0.705 ÷ 0.346)	0.405	13
amateur athletes	0.207 (−0.059 ÷ 0.446)	0.125	56
professional athletes	0.262 (0.088 ÷ 0.421)	0.004 *	121

Note: Statistically significant correlations are indicated with *.

**Table 2:** Correlations between mental resilience and athletes’ age.

Groups of Participants	Spearman’s Rho	*p*
overall sample of athletes	0.107	0.157
athletes in individual sports	0.177	0.105
athletes in combat sports	0.144	0.333
athletes in team sports	0.030	0.846
athletes in swimming	−0.231	0.340
athletes in volleyball	0.054	0.832
track and field athletes	0.132	0.480
athletes in soccer	−0.089	0.772
amateur athletes	0.246	0.068
professional athletes	0.032	0.728

**Table 3:** Correlations between mindfulness and athletes’ age.

Groups of Participants	Spearman’s Rho (with Confidence Intervals)	*p*
overall sample of athletes	0.143 (−0.004 ÷ 0.285)	0.057
athletes in individual sports	−0.036 (−0.247 ÷ 0.178)	0.742
athletes in combat sports	0.369 (0.092 ÷ 0.594)	0.011 *
athletes in team sports	0.310 (0.018 ÷ 0.553)	0.038 *
athletes in swimming	−0.293 (−0.660 ÷ 0.186)	0.223
athletes in volleyball	0.564 (0.132 ÷ 0.816)	0.015 *
track and field athletes	−0.135 (−0.467 ÷ 0.230)	0.469
athletes in soccer	0.227 (−0.370 ÷ 0.692)	0.455
amateur athletes	0.187 (−0.080 ÷ 0.429)	0.167
professional athletes	0.138 (−0.042 ÷ 0.308)	0.133

Note: Statistically significant correlations are indicated with *.

**Table 4:** Correlations between mental resilience and athletes’ years of sports practice.

Groups of Participants	Spearman’s Rho (with Confidence Intervals)	*p*
overall sample of athletes	0.080 (−0.069 ÷ 0.225)	0.292
athletes in individual sports	−0.032 (−0.243 ÷ 0.182)	0.771
athletes in combat sports	0.003 (−0.284 ÷ 0.290)	0.982
athletes in swimming	−0.246 (−0.630 ÷ 0.234)	0.310
athletes in volleyball	0.027 (−0.445 ÷ 0.488)	0.915
athletes in team sports	0.377 (0.094÷0.604)	0.011 *
track and field athletes	−0.056 (−0.402 ÷ 0.305)	0.766
athletes in soccer	0.825 (0.501 ÷ 0.946)	0.001 *
amateur athletes	0.350 (0.096 ÷ 0.561)	0.008 *
professional athletes	−0.081 (−0.256 ÷ 0.099)	0.375

Note: Statistically significant correlations are indicated with *.

**Table 5:** Correlations between mindfulness and athletes’ years of sports practice.

Groups of Participants	Spearman’s Rho (with Confidence Intervals)	*p*
overall sample of athletes	−0.148 (−0.289 ÷ −0.0006)	0.049 *
athletes in individual sports	−0.261 (−0.449 ÷ −0.050)	0.016 *
athletes in combat sports	0.156 (−0.138 ÷ 0.424)	0.296
athletes in swimming	−0.380 (−0.711 ÷ 0.090)	0.108
athletes in volleyball	−0.307 (−0.677 ÷ 0.186)	0.215
athletes in team sports	−0.206 (−0.471 ÷ 0.093)	0.175
track and field athletes	−0.431 (−0.681 ÷ −0.090)	0.016 *
athletes in soccer	−0.222 (−0.689 ÷ 0.375)	0.466
amateur athletes	−0.266 (−0.494 ÷ −0.003)	0.048 *
professional athletes	−0.075 (−0.250 ÷ 0.105)	0.416

Note: Statistically significant correlations are indicated with *.

## Data Availability

Data are available from the corresponding author upon a reasonable request.

## Appendix A

The Benjamini–Hochberg procedure to control the False Discovery Rate (FDR) when conducting multiple statistical correlational tests.

**Table A1:** The Benjamini–Hochberg (BH) procedure to control the False Discovery Rate (FDR) for correlations between mental resilience and mindfulness.

Groups of Participants	*p*-Values Sorted in Ascending Order	BH Threshold i/m⋅α	Comparison of *p*-Values to Their Threshold
overall sample of athletes	0.002	$\frac{1}{10}\times 0.05=0.005$	*p*_1_ = 0.002 ≤ 0.005 → Reject the first null hypothesis
professional athletes	0.004	$\frac{2}{10}\times 0.05=0.01$	*p*_2_ = 0.004 ≤ 0.010 → Reject the second null hypothesis
athletes practicing individual sports	0.019	$\frac{3}{10}\times 0.05=0.015$	*p*_3_ = 0.019 > 0.015 → Do not reject the third null hypothesis
athletes practicing swimming	0.023	$\frac{4}{10}\times 0.05=0.02$	*p*_4_ = 0.023 > 0.020 → Do not reject the fourth null hypothesis
athletes practicing volleyball	0.026	$\frac{5}{10}\times 0.05=0.025$	*p*_5_ = 0.026 > 0.025 → Do not reject the fifth null hypothesis
athletes practicing combat sports	0.033	$\frac{6}{10}\times 0.05=0.03$	*p*_6_ = 0.033 > 0.030 → Do not reject the sixth null hypothesis
athletes practicing team sports	0.061	$\frac{7}{10}\times 0.05=0.035$	*p*_7_ = 0.061 > 0.035 → Do not reject the seventh null hypothesis
amateur athletes	0.125	$\frac{8}{10}\times 0.05=0.04$	*p*_8_ = 0.125 > 0.040 → Do not reject the eighth null hypothesis
athletes practicing soccer	0.405	$\frac{9}{10}\times 0.05=0.045$	*p*_9_ = 0.405 > 0.045 → Do not reject the ninth null hypothesis
track and field athletes	0.882	$\frac{10}{10}\times 0.05=0.05$	*p*_10_ = 0.882 > 0.050 → Do not reject the tenth null hypothesis

Note: i means the rank order of the hypothesis; m means the number of hypotheses; the desired False Discovery Rate level α was 0.05. → means therefore, consequently.

**Table A2:** The Benjamini–Hochberg (BH) procedure to control the False Discovery Rate (FDR) for correlations between mindfulness and athletes’ age.

Groups of Participants	*p*-Values Sorted in Ascending Order	BH Threshold i/m⋅α	Comparison of *p*-Values to Their Threshold
athletes practicing combat sports	0.011	$\frac{1}{10}\times 0.05=0.005$	*p*_1_ = 0.011 > 0.005 → Do not reject the first null hypothesis
athletes practicing volleyball	0.015	$\frac{2}{10}\times 0.05=0.01$	*p*_2_ = 0.015 > 0.010 → Do not reject the second null hypothesis
athletes practicing team sports	0.038	$\frac{3}{10}\times 0.05=0.015$	*p*_3_ = 0.038 > 0.015 → Do not reject the third null hypothesis
overall sample of athletes	0.057	$\frac{4}{10}\times 0.05=0.02$	*p*_4_ = 0.057 > 0.020 → Do not reject the fourth null hypothesis
professional athletes	0.133	$\frac{5}{10}\times 0.05=0.025$	*p*_5_ = 0.133 > 0.025 → Do not reject the fifth null hypothesis
amateur athletes	0.167	$\frac{6}{10}\times 0.05=0.03$	*p*_6_ = 0.167 > 0.030 → Do not reject the sixth null hypothesis
athletes practicing swimming	0.223	$\frac{7}{10}\times 0.05=0.035$	*p*_7_ = 0.223 > 0.035 → Do not reject the seventh null hypothesis
athletes practicing soccer	0.455	$\frac{8}{10}\times 0.05=0.04$	*p*_8_ = 0.455 > 0.040 → Do not reject the eighth null hypothesis
athletes practicing track and field athletics	0.469	$\frac{9}{10}\times 0.05=0.045$	*p*_9_ = 0.469 > 0.045 → Do not reject the ninth null hypothesis
athletes practicing individual sports	0.742	$\frac{10}{10}\times 0.05=0.05$	*p*_10_ = 0.742 > 0.050 → Do not reject the tenth null hypothesis

Note: i means the rank order of the hypothesis; m means the number of hypotheses; the desired False Discovery Rate level α was 0.05. → means therefore, consequently.

**Table A3:** The Benjamini–Hochberg (BH) procedure to control the False Discovery Rate (FDR) for correlations between mental resilience and athletes’ years of sports practice.

Groups of Participants	*p*-Values Sorted in Ascending Order	BH Threshold i/m⋅α	Comparison of *p*-Values to Their Threshold
athletes practicing soccer	0.001	$\frac{1}{10}\times 0.05=0.005$	*p*_1_ = 0.001 ≤ 0.005 → Reject the first null hypothesis
amateur athletes	0.008	$\frac{2}{10}\times 0.05=0.01$	*p*_2_ = 0.008 ≤ 0.010 → Reject the second null hypothesis
athletes practicing team sports	0.011	$\frac{3}{10}\times 0.05=0.015$	*p*_3_ = 0.011 ≤ 0.015 → Reject the third null hypothesis
overall sample of athletes	0.292	$\frac{4}{10}\times 0.05=0.02$	*p*_4_ = 0.292 > 0.020 → Do not reject the fourth null hypothesis
athletes practicing swimming	0.310	$\frac{5}{10}\times 0.05=0.025$	*p*_5_ = 0.310 > 0.025 → Do not reject the fifth null hypothesis
professional athletes	0.375	$\frac{6}{10}\times 0.05=0.03$	*p*_6_ = 0.375 > 0.030 → Do not reject the sixth null hypothesis
track and field athletes	0.766	$\frac{7}{10}\times 0.05=0.035$	*p*_7_ = 0.766 > 0.035 → Do not reject the seventh null hypothesis
athletes practicing individual sports	0.771	$\frac{8}{10}\times 0.05=0.04$	*p*_8_ = 0.771 > 0.040 → Do not reject the eighth null hypothesis
athletes practicing volleyball	0.915	$\frac{9}{10}\times 0.05=0.045$	*p*_9_ = 0.915 > 0.045 → Do not reject the ninth null hypothesis
athletes practicing combat sports	0.982	$\frac{10}{10}\times 0.05=0.05$	*p*_10_ = 0.982 > 0.050 → Do not reject the tenth null hypothesis

Note: i means the rank order of the hypothesis; m means the number of hypotheses; the desired False Discovery Rate level α was 0.05. → means therefore, consequently.

**Table A4:** The Benjamini–Hochberg (BH) procedure to control the False Discovery Rate (FDR) for correlations between mindfulness and athletes’ years of sports practice.

Groups of Participants	*p*-Values Sorted in Ascending Order	BH Threshold i/m⋅α	Comparison of *p*-Values to Their Threshold
athletes practicing individual sports	0.016	$\frac{1}{10}\times 0.05=0.005$	*p*_1_ = 0.016 > 0.005 → Do not reject the first null hypothesis
track and field athletes	0.016	$\frac{2}{10}\times 0.05=0.01$	*p*_2_ = 0.016 > 0.010 → Do not reject the second null hypothesis
amateur athletes	0.048	$\frac{3}{10}\times 0.05=0.015$	*p*_3_ = 0.048 > 0.015 → Do not reject the third null hypothesis
overall sample of athletes	0.049	$\frac{4}{10}\times 0.05=0.02$	*p*_4_ = 0.049 > 0.020 → Do not reject the fourth null hypothesis
athletes practicing swimming	0.108	$\frac{5}{10}\times 0.05=0.025$	*p*_5_ = 0.108 > 0.025 → Do not reject the fifth null hypothesis
athletes practicing team sports	0.175	$\frac{6}{10}\times 0.05=0.03$	*p*_6_ = 0.175 > 0.030 → Do not reject the sixth null hypothesis
athletes practicing volleyball	0.215	$\frac{7}{10}\times 0.05=0.035$	*p*_7_ = 0.215 > 0.035 → Do not reject the seventh null hypothesis
athletes practicing combat sports	0.296	$\frac{8}{10}\times 0.05=0.04$	*p*_8_ = 0.296 > 0.040 → Do not reject the eighth null hypothesis
professional athletes	0.416	$\frac{9}{10}\times 0.05=0.045$	*p*_9_ = 0.416 > 0.045 → Do not reject the ninth null hypothesis
athletes practicing soccer	0.466	$\frac{10}{10}\times 0.05=0.05$	*p*_10_ = 0.466 > 0.050 → Do not reject the tenth null hypothesis

Note: i means the rank order of the hypothesis; m means the number of hypotheses; the desired False Discovery Rate level α was 0.05. → means therefore, consequently.

## Appendix B

Application of the Holm–Bonferroni method to deal with familywise error rates (FWER) for multiple statistical correlational tests.

**Table A5:** The Holm–Bonferroni method to adjust for multiple comparisons controlling family-wise error for correlations between mental resilience and mindfulness.

Groups of Participants	*p*-Values Sorted in Ascending Order	Holm–Bonferroni Formula HB = Target α/(*n* − Rank + 1)	Comparison of *p*-Values to Their Threshold
overall sample athletes	0.002	0.05/(10 − 1 + 1) = 0.005	*p*_1_ = 0.002 ≤ 0.005 → Reject the first null hypothesis
professional athletes	0.004	0.05/(10 − 2 + 1) = 0.0055	*p*_2_ = 0.004 ≤ 0.0055 → Reject the second null hypothesis
athletes practicing individual sports	0.019	0.05/(10 − 3 + 1) = 0.00625	*p*_3_ = 0.019 > 0.00625 → Do not reject the third null hypothesis
athletes practicing swimming	0.023	0.05/(10 − 4 + 1) = 0.0071	*p*_4_ = 0.023 > 0.0071 → Do not reject the fourth null hypothesis
athletes practicing volleyball	0.026	0.05/(10 − 5 + 1) = 0.0083	*p*_5_ = 0.026 > 0.0083 → Do not reject the fifth null hypothesis
athletes practicing combat sports	0.033	0.05/(10 − 6 + 1) = 0.01	*p*_6_ = 0.033 > 0.010 → Do not reject the sixth null hypothesis
athletes practicing team sports	0.061	0.05/(10 − 7 + 1) = 0.0125	*p*_7_ = 0.061 > 0.0125 → Do not reject the seventh null hypothesis
amateur athletes	0.125	0.05/(10 − 8 + 1) = 0.0166	*p*_8_ = 0.125 > 0.0166 → Do not reject the eighth null hypothesis
athletes practicing soccer	0.405	0.05/(10 − 9 + 1) = 0.025	*p*_9_ = 0.405 > 0.025 → Do not reject the ninth null hypothesis
track and field athletes	0.882	0.05/(10 − 10 + 1) = 0.05	*p*_10_ = 0.882 > 0.050 → Do not reject the tenth null hypothesis

Note: *n* means the number of hypotheses; the target level α was 0.05. → means therefore, consequently.

**Table A6:** The Holm–Bonferroni method to adjust for multiple comparisons controlling family-wise error for correlations between mindfulness and athletes’ age.

Groups of Participants	*p*-Values Sorted in Ascending Order	Holm–Bonferroni Formula HB = Target α/(*n* − Rank + 1)	Comparison of *p*-Values to Their Threshold
athletes practicing combat sports	0.011	0.05/(10 − 1 + 1) = 0.005	*p*_1_ = 0.011 > 0.005 → Do not reject the first null hypothesis
athletes practicing volleyball	0.015	0.05/(10 − 2 + 1) = 0.0055	*p*_2_ = 0.015 > 0.0055 → Do not reject the second null hypothesis
athletes practicing team sports	0.038	0.05/(10 − 3 + 1) = 0.00625	*p*_3_ = 0.038 > 0.00625 → Do not reject the third null hypothesis
overall sample of athletes	0.057	0.05/(10 − 4 + 1) = 0.0071	*p*_4_ = 0.057 > 0.0071 → Do not reject the fourth null hypothesis
professional athletes	0.133	0.05/(10 − 5 + 1) = 0.0083	*p*_5_ = 0.133 > 0.0083 → Do not reject the fifth null hypothesis
amateur athletes	0.167	0.05/(10 − 6 + 1) = 0.01	*p*_6_ = 0.167 > 0.010 → Do not reject the sixth null hypothesis
athletes practicing swimming	0.223	0.05/(10 − 7 + 1) = 0.0125	*p*_7_ = 0.223 > 0.0125 → Do not reject the seventh null hypothesis
athletes practicing soccer	0.455	0.05/(10 − 8 + 1) = 0.0166	*p*_8_ = 0.455 > 0.0166 → Do not reject the eighth null hypothesis
track and field athletes	0.469	0.05/(10 − 9 + 1) = 0.025	*p*_9_ = 0.469 > 0.025 → Do not reject the ninth null hypothesis
athletes practicing individual sports	0.742	0.05/(10 − 10 + 1) = 0.05	*p*_10_ = 0.742 > 0.050 → Do not reject the tenth null hypothesis

Note: *n* means the number of hypotheses; the target level α was 0.05. → means therefore, consequently.

**Table A7:** The Holm–Bonferroni method to adjust for multiple comparisons controlling family-wise error for correlations between mental resilience and athletes’ years of sports practice.

Groups of Participants	*p*-Values Sorted in Ascending Order	Holm–Bonferroni Formula HB = Target α/(*n* − Rank + 1)	Comparison of *p*-Values to Their Threshold
athletes practicing soccer	0.001	0.05/(10 − 1 + 1) = 0.005	*p*_1_ = 0.001 ≤ 0.005 → Reject the first null hypothesis
amateur athletes	0.008	0.05/(10 − 2 + 1) = 0.0055	*p*_2_ = 0.008 > 0.0055 → Do not reject the second null hypothesis
athletes practicing team sports	0.011	0.05/(10 − 3 + 1) = 0.00625	*p*_3_ = 0.011 > 0.00625 → Do not reject the third null hypothesis
overall sample of athletes	0.292	0.05/(10 − 4 + 1) = 0.0071	*p*_4_ = 0.292 > 0.0071 → Do not reject the fourth null hypothesis
athletes practicing swimming	0.310	0.05/(10 − 5 + 1) = 0.0083	*p*_5_ = 0.310 > 0.0083 → Do not reject the fifth null hypothesis
professional athletes	0.375	0.05/(10 − 6 + 1) = 0.01	*p*_6_ = 0.375 > 0.010 → Do not reject the sixth null hypothesis
track and field athletes	0.766	0.05/(10 − 7 + 1) = 0.0125	*p*_7_ = 0.766 > 0.0125 → Do not reject the seventh null hypothesis
athletes practicing individual sports	0.771	0.05/(10 − 8 + 1) = 0.0166	*p*_8_ = 0.771 > 0.0166 → Do not reject the eighth null hypothesis
athletes practicing volleyball	0.915	0.05/(10 − 9 + 1) = 0.025	*p*_9_ = 0.915 > 0.025 → Do not reject the ninth null hypothesis
athletes practicing combat sports	0.982	0.05/(10 − 10 + 1) = 0.05	*p*_10_ = 0.982 > 0.050 → Do not reject the tenth null hypothesis

Note: *n* means the number of hypotheses; the target level α was 0.05. → means therefore, consequently.

**Table A8:** The Holm–Bonferroni method to adjust for multiple comparisons controlling family-wise error for correlations between mindfulness and athletes’ years of sports practice.

Groups of Participants	*p*-Values Sorted in Ascending Order	Holm–Bonferroni Formula HB = Target α/(*n* − Rank + 1)	Comparison of *p*-Values to Their Threshold
athletes practicing individual sports	0.016	0.05/(10 − 1 + 1) = 0.005	*p*_1_ = 0.016 > 0.005 → Do not reject the first null hypothesis
track and field athletes	0.016	0.05/(10 − 2 + 1) = 0.0055	*p*_2_ = 0.016 > 0.0055 → Do not reject the second null hypothesis
amateur athletes	0.048	0.05/(10 − 3 + 1) = 0.00625	*p*_3_ = 0.048 > 0.00625 → Do not reject the third null hypothesis
overall sample of athletes	0.049	0.05/(10 − 4 + 1) = 0.0071	*p*_4_ = 0.049 > 0.0071 → Do not reject the fourth null hypothesis
athletes practicing swimming	0.108	0.05/(10 − 5 + 1) = 0.0083	*p*_5_ = 0.108 > 0.0083 → Do not reject the fifth null hypothesis
athletes practicing team sports	0.175	0.05/(10 − 6 + 1) = 0.01	*p*_6_ = 0.175 > 0.010 → Do not reject the sixth null hypothesis
athletes practicing volleyball	0.215	0.05/(10 − 7 + 1) = 0.0125	*p*_7_ = 0.215 > 0.0125 → Do not reject the seventh null hypothesis
athletes practicing combat sports	0.296	0.05/(10 − 8 + 1) = 0.0166	*p*_8_ = 0.296 > 0.0166 → Do not reject the eighth null hypothesis
professional athletes	0.416	0.05/(10 − 9 + 1) = 0.025	*p*_9_ = 0.416 > 0.025 → Do not reject the ninth null hypothesis
athletes practicing soccer	0.466	0.05/(10 − 10 + 1) = 0.05	*p*_10_ = 0.466 > 0.050 → Do not reject the tenth null hypothesis

Note: *n* means the number of hypotheses; the target level α was 0.05. → means therefore, consequently.
